# A Mixed-Methods Systematic Review: Infidelity, Romantic Jealousy and Intimate Partner Violence against Women

**DOI:** 10.3390/ijerph17165682

**Published:** 2020-08-06

**Authors:** Marjorie Pichon, Sarah Treves-Kagan, Erin Stern, Nambusi Kyegombe, Heidi Stöckl, Ana Maria Buller

**Affiliations:** 1 Department of Global Health and Development, London School of Hygiene and Tropical Medicine, London WC1H 9SH, UK; Erin.Stern@lshtm.ac.uk (E.S.); Nambusi.Kyegombe@lshtm.ac.uk (N.K.); Heidi.Stoeckl@lshtm.ac.uk (H.S.); Ana.Buller@lshtm.ac.uk (A.M.B.); 2 Department of Health Behavior, Gillings School of Global Health, University of North Carolina, Chapel Hill, NC 27599, USA; Kagan.sarah@gmail.com

**Keywords:** intimate partner violence, domestic violence, family violence, spouse abuse, controlling behaviour, infidelity, unfaithfulness, romantic jealousy, gender, systematic review

## Abstract

Infidelity and romantic jealousy (RJ) are commonly cited relational level drivers of intimate partner violence (IPV) but remain undertheorized and underutilized in IPV research and prevention. This global systematic review aims to characterize the existing research on real or suspected infidelity and RJ in relation to IPV and inform future research and programming. We systematically searched 11 databases for peer-reviewed research, published between April 2009 and 2019, that provided data on the prevalence or a measure of association (quantitative), or pathway (qualitative), between real or suspected infidelity or RJ, and IPV. Fifty-one papers from 28 countries were included and the evidence showed a consistent association between real or suspected infidelity, RJ and IPV. Our findings identify three overarching mechanisms and six pathways between infidelity, RJ and IPV. These provide support for prominent theories in the field related to patriarchal culture, threatened masculinities and femininities and a lack of emotional regulation and conflict resolution skills, but not evolutionary theories. Our findings suggest that researchers should use standardized measurement tools that make the distinction between RJ and suspected, confirmed and accusations of infidelity. Policy and programming should aim to transform traditional gender roles, accounting for infidelity and RJ and improving couple’s communication and trust.

## 1. Introduction

Globally, an estimated one-fourth of women are expected to experience a form of intimate partner violence (IPV) in their lifetime [1]. IPV can lead to a wide-range of negative health impacts including depression, alcohol use disorder, low-birth rate, sexually transmitted infections (STIs), injury and death [2], with over a third of female homicides perpetrated by an intimate partner [3].

### 1.1. Defining Terminology

The World Health Organization (WHO) [4] identifies four types of IPV: (1) physical violence—including degrees of severity from slapping to homicide; (2) sexual violence—including forced sex and sexual coercion; (3) psychological violence—including insults, humiliation and threats and (4) controlling behaviours—including isolating a person from family and friends, monitoring their movements or restricting their access to financial resources, employment, education or medical care. Economic violence is a frequently cited fifth type of IPV that warrants separate investigation, distinct from general controlling behaviours; it occurs when one is prevented from being economically independent, for example by being prevented from gaining employment, having earnings taken or being forced from one’s home [5].

Infidelity is defined as “(the act of) having sex with someone who is not your husband, wife, or regular sexual partner” [6]. A partner’s real or suspected infidelity may—or may not—cause romantic jealousy (RJ), which is described by White [7] as “a complex set of thoughts, feelings and actions that follow a threat to self-esteem and/or threaten the existence or quality of the relationship”. The term RJ is used to distinguish it from other types of jealousy, such as sibling rivalry or jealousy that occurs between an adult and child [8]. RJ is usually conceptualized as an amalgamation of various emotions—that can differ based on cultural context, among other factors—and include, but are not limited to: anger, frustration, insecurity, unluckiness, helplessness, sadness, grief, shame, embarrassment and humiliation [8,9]. Differences across cultures may also exist in how RJ is provoked, how often RJ is felt, how legitimate feelings of RJ are thought to be (and whether it is more appropriate to discuss infidelity instead of RJ) and which behaviours are considered typical in response to RJ [10].

### 1.2. Relational Level Drivers of Intimate Partner Violence

The ecological framework for understanding violence against women conceptualizes IPV as originating at the individual, relational, community and structural levels, and it is essential to tackle drivers at all of these levels to successfully reduce IPV [4,11,12,13]. The relational level accounts for factors that affect how the couple interacts with one another—thereby impacting their relationship—for example, male dominance in the family, male control of wealth, alcohol use and marital conflict [11]; including conflicts that arise from real or suspected infidelity and RJ. A recent review identified poor communication and conflict in relationships as a major driver of IPV, emphasizing the continued relevance of the relational level [14]. However, real or suspected infidelity and RJ remain understudied, undertheorized and underutilized in IPV prevention efforts.

Findings from a recent systematic review on RJ in relationships, and studies on women’s experiences of IPV, indicate strong evidence of association between male RJ and physical, psychological and sexual male-to-female IPV (referred to as IPV here-on-out unless otherwise specified) [15,16,17]. Bidirectional psychological IPV has also been found to be associated with higher levels of dominance and RJ in both men and women [18,19]. Additional findings from limited research suggest that anxiety and depression may play a role in the association between partner infidelity and IPV [20,21]. For example, symptoms of anxiety have been found to mediate the relationship between men’s anticipated partner infidelity and IPV [22].

The relevance of relational level drivers is also highlighted in interventions that have been successful in reducing IPV. Interventions in Ecuador and Uganda for example, specifically found that decreasing men’s suspicion of partner infidelity played a major role in their success [23,24]. Societal level factors such as the belief that RJ is desirable in relationships [25], patriarchal norms [26] and social acceptance of IPV [27] may also moderate the relationship between real or suspected infidelity, RJ and IPV.

Despite these findings, there has been limited research conducted on infidelity, RJ and IPV. The aim of this review is to characterize and synthesize the existing literature, elucidating the frequency with which infidelity and RJ are cited as triggers for IPV across different regions, how widespread the association between them is, and the mechanisms and pathways of infidelity and RJ leading to IPV. Ultimately, this review expands the knowledge base on relational level drivers of IPV, identifying gaps in the literature and recommendations for programming and research.

## 2. Methods

### 2.1. Design and Search Strategy

We systematically searched 11 medical and social sciences databases (ASSIA (Applied Social Sciences Index and Abstracts), CENTRAL (Cochrane Central Register of Controlled Trials), CINAHL (Cumulative Index of Nursing and Allied Health Literature), Embase (Excerpta Medica Database), IBSS (International Bibliography of the Social Sciences), Medline (Medical Literature Analysis and Retrieval System Online), PsycINFO (Psychological Information), Social Policy and Practice, Social Services Abstracts, Sociological Abstracts and Web of Science) with key search terms related to IPV adapted from Cochrane protocols [28,29], and to infidelity or RJ adapted from previous reviews [15,30,31], and a scoping review of the literature (see Appendix A for our full search strategy). This review protocol is registered with the PROSPERO (International Prospective Register of Systematic Reviews) database (registration ID: CRD42019130697). The search was conducted on the 10th of April 2019 and was limited to peer-reviewed results published since the 10th of April 2009 in English, French, German, Italian and Portuguese, reflecting the language expertise of the authors.

### 2.2. Inclusion and Exclusion Criteria

To be eligible for inclusion, studies had to: (1) provide data on prevalence or measure of association (quantitative), or pathway (qualitative), between infidelity or RJ in male-to-female IPV, (2) focus on adults 18 years and older (studies that sampled adolescents and adults were only included if they provide disaggregated data on adults 18 years and older) and (3) include participants in any kind of current, heterosexual partnership (including dating, cohabitating or married), and provide disaggregated results for them.

Studies were excluded if they: (1) did not base their findings on empirical research (e.g., commentaries or theoretical papers) or were case studies or grey literature, (2) sampled university students or people with diagnosed medical conditions (e.g., depression, personality disorders and pathological jealousy), (3) measured predicted or feared IPV, or infidelity or RJ as part of a bigger variable and did not report disaggregated findings for them or (4) used proxy’s for infidelity or RJ such as “women who have a child by another man” (see Table 1).

### 2.3. Screening

**Figure 1:**
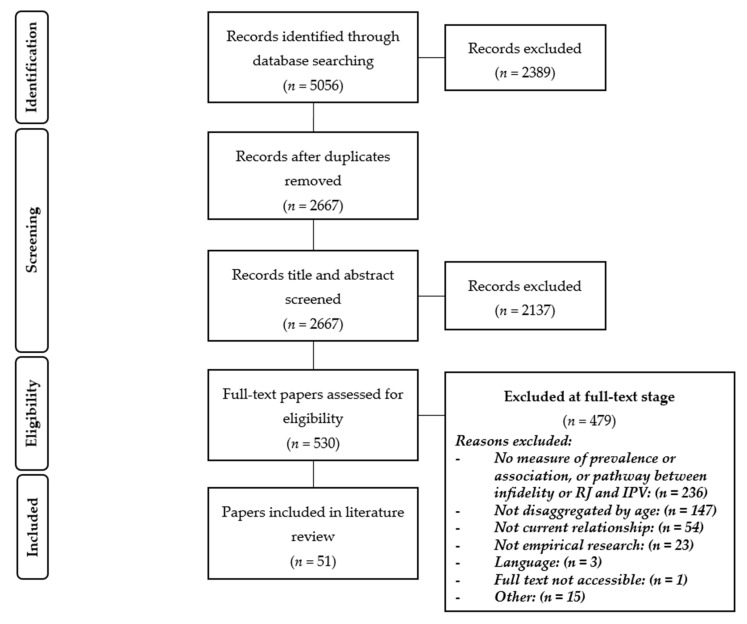
PRISMA 2009 flowchart of the study selection process.

Following PRISMA (preferred reporting items for systematic reviews and meta-analyses) guidelines [45] we first screened the titles and abstracts of papers identified in our search (see Figure 1). All titles and abstracts were screened by Marjorie Pichon (MP), and 20% were dual-screened by Sarah Treves-Kagan (STK), Nambusi Kyegombe (NK), and Ana Maria Buller (AMB) to measure a concordance rate of above 80% between MP and all other authors. All papers that were not excluded were then full-text screened by MP, and 20% were dual screened by STK, NK, Heidi Stöckl (HS) and AMB. A concordance rate of over 85% was achieved between them and MP at this stage. Disagreements in both stages were discussed until consensus was reached.

### 2.4. Data Extraction and Analyses

Authors MP, STK, HS and AMB extracted data from quantitative studies using a customized excel spreadsheet that included: sample characteristics, study design, measures of infidelity or RJ and IPV used, findings of prevalence and association and explanations provided by authors of their results. Authors dual-extracted 30% of papers to ensure consistency. Findings of prevalence and association were organized by region and compared, and explanations provided by authors of their results were thematically analysed.

MP, Erin Stern (ES), NK and AMB coded and analysed qualitative studies using a deductive and inductive approach supported by NVivo 12. MP first created a coding framework based on the preliminary coding of 15 data-rich papers. All authors then independently coded the same paper and discussed findings, refining the coding framework before independently coding the remaining included papers. MP then reviewed all coding and standardized discrepancies.

### 2.5. Quality Appraisal

Quantitative studies were quality appraised using criteria adapted from a validated tool [46] and the STROBE (Strengthening the Reporting of Observational Studies in Epidemiology) checklist [47], while qualitative studies were quality appraised using criteria adapted from the CASP (Critical Appraisal Skills Programme) [48] checklist. Thirty percent of all studies were dual-appraised to ensure consistency, and a concordance rate of 75% was achieved between MP and all other authors. When disagreements arose authors discussed until a consensus was reached.

## 3. Results

### 3.1. Study Characteristics

A total of 51 papers met our inclusion criteria. These papers were derived from 50 studies, 25 of which used quantitative methods and 25 of which used qualitative methods. They included evidence from 28 countries, covering all world regions; the most well represented were the USA (*n =* 14), Turkey (*n =* 4), India (*n =* 3) and South Africa (*n =* 3). Most participants were married or living with a long-term partner. Physical IPV was the most common form of IPV studied in relation to infidelity or RJ, while economic IPV was the least common.

### 3.2. Summary of Included Quantitative Studies

The sample size of included quantitative studies ranged from 43 to 5000. Quality assessment revealed eight high-quality studies (three or four stars) and 17 low-quality studies (one or two stars). The main reasons studies received low scores were because they were not representative of the population, the missing data policy was not described or the missing data were excluded without a sensitivity analysis being conducted, or a non-response bias assessment was not described (see Table 2, and Appendix B for the full quality assessment). The most well represented regions were North America (*n =* 10) and Europe and Central Asia (*n =* 4), and almost all studies were cross-sectional in design (*n =* 22) and used random or convenience sampling (see Table 3).

### 3.3. Measurement of Infidelity and RJ

Only 32% of studies used a validated questionnaire or scale to measure real or suspected infidelity or RJ (*n =* 8) including the negotiation subscale of the revised Conflict Tactic Scale (CTS2) [75], the multidimensional jealousy scale [76] and the relationship jealousy scale [77]. The most common infidelity or RJ outcomes measured were male RJ (*n =* 12) and male infidelity (*n =* 7). By contrast, female infidelity (*n =* 2) and female suspicion of male infidelity (*n =* 4) were rarely measured. There were a wide range of questions used to capture infidelity or RJ, from direct questions about behaviour “since you have been together, has your spouse or steady partner had sex with another partner?” [72] to open questions about causes of violence “what are the reasons for domestic violence given by your husband?” [49] (see Appendix C for a description of all measures of infidelity and RJ used in included studies).

### 3.4. Measurement of IPV

The most common IPV measurement instruments used were the CTS or a revision or adaptation of it (*n =* 9), or an inventory of specific behaviours, such as pushing or shoving (*n =* 6). Most studies measured physical IPV only, including intimate partner femicide (*n =* 8), or a combination of physical, sexual (including sexual coercion), and psychological IPV (*n =* 6). No studies measured psychological or economic IPV independently, and only two studies measured economic IPV in addition to measuring all other types. IPV reporters were most often women (*n =* 15), and less commonly couples (*n =* 5) or men (*n =* 3).

### 3.5. Frequency of Infidelity or RJ as a Reason for IPV

In total, 12 studies reported how many women or men cited real or suspected infidelity or RJ as the reason for their experience or perpetration of IPV. In North and Latin America, infidelity or RJ emerged as the leading reasons. A study in Canada found that 45% of men reported their RJ as a reason for their perpetration of physical or sexual IPV [57], while in the USA 59% of men in a perpetrator intervention program reported female RJ as a reason for their perpetration of physical or psychological IPV [60]. In Mexico, 48% of sampled female sex workers cited male infidelity by a steady partner as a reason they had experienced physical, sexual or psychological IPV [72].

In studies from Europe and Central Asia, South Asia and East Asia and the Pacific the frequency of infidelity or RJ reported as a reason for IPV was lower, but still one of the most commonly cited. In Turkey male RJ was cited by women as the most common reason—ranging from 18% [50] to 33% [49]—for physical, sexual, psychological or economic IPV, while a study in the Philippines reported that 17% of participants cited RJ (male or female) as responsible for physical, psychological or sexual IPV; the most common triggers behind alcohol consumption and “nagging” [51]. In Nepal, 11% of sampled pregnant women cited male infidelity as a reason they had experienced physical, sexual or psychological IPV [66].

Infidelity or RJ was cited more frequently, however, as motives in cases of intimate partner homicide in these regions. A study among Ethiopian immigrants in Israel found that male RJ was the second most common motivation for murder after separation—ranging from 14% for those who strangled their partner, to 57% for those who stabbed their partner—and was the most common motive cited for homicides committed with excessive injury [54]. Data from Turkey found infidelity, RJ or honour killings as a motivation behind 45% of investigated intimate partner homicides, but who had been unfaithful or was jealous was not specified [70].

### 3.6. IPV Outcomes and Associations with Infidelity or RJ

Of the 25 included quantitative studies, 10 measured unadjusted odds ratios or bivariate correlations between real or suspected infidelity or RJ and IPV, and 14 measured adjusted odds ratios or multivariate correlations (some measured both). Of these, 19 found that experiences of infidelity or RJ significantly increased women’s likelihood of experiencing IPV, five found no association and none found a negative association.

In studies from North and Latin America the association was particularly strong for physical and sexual IPV and RJ. In several US studies, male RJ was found to be associated with physical IPV [59,62], sexual IPV [67] and physical or sexual IPV [60], while mutual RJ by both partners was found to be associated with physical IPV, and this association was greater among reciprocally violent couples [61].

While the overall associations were strong between infidelity or RJ and IPV in these regions, several studies provided more nuanced findings, by differentiating according to ethnicity or types of IPV. For example, male infidelity was found to be associated with physical IPV in participants of Mexican origin but not Puerto Rican origin in the USA [64], suspicion of infidelity (male or female) and female RJ were found to not be associated with physical IPV [62], and while male RJ was found to be associated with forced sex, it was not associated with other forms of sexual coercion or abuse [63]. Sexual coercion was found to be associated with both real or suspected female infidelity [55] and male and female RJ [65]. In non-representative samples from Bolivia and Mexico, male infidelity was found to be associated with physical IPV [68,69], and physical, sexual or psychological IPV [72].

In studies from Europe and Central Asia, South Asia and East Asia and the Pacific, the association was particularly strong for physical IPV, which was found to be associated with male suspicion of female infidelity [52], male RJ [51], female RJ [73] and mutual RJ by both partners [73]. Female suspicion of male infidelity [66] and male RJ [58] and were also found to be associated with physical, sexual or psychological IPV.

Only two included quantitative studies were conducted in sub-Saharan Africa. A study in rural Malawi found that women were more likely to experience physical IPV and sexual coercion if either partner suspected the other of infidelity, but did not find an association between violence and self-reported marital infidelity [53]; while a study with men that had multiple, concurrent sexual relationships in South Africa found that suspected female infidelity was associated with physical IPV only, physical and sexual IPV, but not sexual IPV only [71]. Male infidelity was also found to be associated with sexual IPV, but not physical IPV only or combined physical or sexual IPV [71].

### 3.7. Underlying Mechanisms of Association between Infidelity, RJ and IPV

There were four mechanisms described by the authors of included quantitative studies to understand the relationship between real or suspected infidelity or RJ and IPV (see Table 3). The most common explanation related to a culture of patriarchy, which maintained that traditional gender norms of male dominance and associated control, and female subservience and the limitation of women’s movements and opportunities outside the home, were the underlying reason for the connection between infidelity and RJ, and IPV. In addition to promoting male possessiveness and RJ as masculine behaviour, the patriarchy also comes with a greater social acceptance of male infidelity and IPV.

Four studies argued that IPV related to infidelity or RJ could be explained by threatened masculinities (see Table 3). These explanations suggest that when traditional patriarchal gender norms are threatened by women questioning male infidelity, being unfaithful themselves or gaining employment, men feel that their achievement of hegemonic masculinities—which is often centred around sexual conquest, dominance and being the financial provider for the family—is threatened. This can lead to male feelings of dependence or impotence, and men blaming their partners for evoking these feelings. Men in turn may respond with IPV to punish their partner, in order to re-establish their dominance and the gendered hierarchy they are accustomed to. The majority of studies that used the patriarchal culture or threatened masculinities explanations were conducted in the Central and South Asian regions.

Seven studies, predominantly in the USA, relied on explanations related to the lack of emotional regulation and conflict resolution skills (see Table 3). These studies suggested that when conflicts related to infidelity and RJ arise in a relationship, some couples have poor conflict resolution skills. Men in these relationships seek greater control of their partner to manage the conflict, or they are unable to control their emotional reactions at all, and the conflict escalates to IPV.

Five studies conducted in North and Latin America or Europe took an evolutionary or biological perspective (see Table 3). These studies argued that men are biologically motivated to have as many partners as possible, and to protect their sexual partners from male competition; they may be motivated to be sexually coercive—especially if they suspect partner infidelity—to introduce their sperm and increase chances of reproduction. They may also be motivated to use physical IPV to end a pregnancy if they have paternity doubts, or to manage partner protests to infidelity.

### 3.8. Summary of Qualitative Results

The sample size of the 26 included qualitative papers ranged from 7 to 95. The most well represented regions were sub-Saharan Africa (*n =* 7), North America (*n =* 5), Latin America and Caribbean (*n =* 4) and South Asia (*n =* 4). Physical IPV was the most common type of IPV studied (*n =* 24) followed by psychological IPV (*n =* 12). Few studies investigated sexual IPV (*n =* 6) or economic IPV (*n =* 6) in relation to real or suspected infidelity or RJ.

Of the papers included, 18 were rated as high-quality (three or four stars), and 8 were rated low-quality (one or two stars). The most common reasons that studies scored poorly were because the authors did not adequately describe how they took into consideration the relationship between the researchers and participants, or ethical issues (such as informed consent and confidentiality), or because they did not clearly outline a data analysis process that explained how quotes were selected and prioritized (see Table 4, and Appendix D for the full quality assessment). The most common methods of analysis used were thematic and content analysis. One study did not analyse data at all, but simply summarized case files.

### 3.9. Identified Mechanisms and Pathways from Infidelity and RJ to IPV

**Figure 2:**
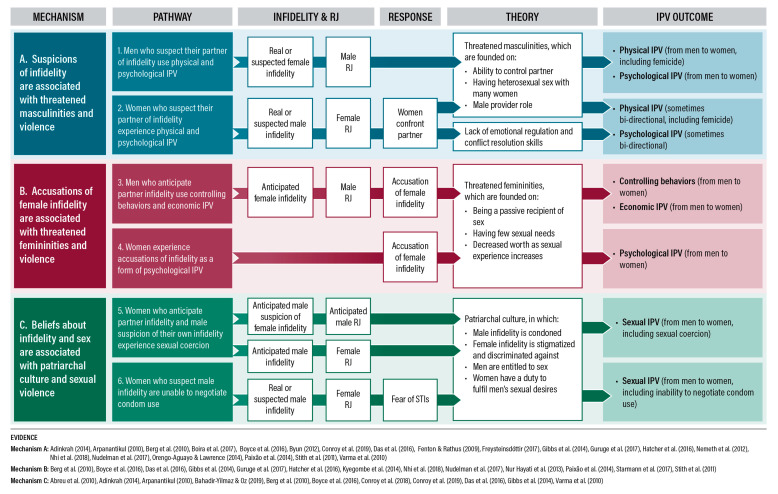
Identified mechanisms and pathways from infidelity and romantic jealousy (RJ) to intimate partner violence (IPV).

Qualitatively, three overarching mechanisms with two pathways within each mechanism emerged, providing a total of six pathways that further explain the association between real or suspected infidelity and RJ, and IPV (see Figure 2). We describe these pathways narratively, highlighting how participants may move fluidly between them, and reporting the related triggers, mechanisms and cultural norms that emerged from the literature.

#### 3.9.1. Mechanism A: Suspicions of Infidelity Are Associated with Threatened Masculinities and Violence

**Pathway** **1.** Men who suspect their partner of infidelity use physical and psychological IPV.

In studies from across the world participants reported that women who were suspected of infidelity experienced physical IPV ranging from hitting, slapping and biting [85,92,95,100], to violence that made them fear for their lives, such as being punched, suffocated, locked up and having a gun put to their head [93,95,99,100].

These acts of violence were described by participants as usually occurring as a reaction to triggering events, such as a woman coming home later than expected [93,95], or her partner seeing her speaking with another man [97,99]; triggers that constituted a direct threat to aspired masculinities, which were centred on a man’s ability to control his partner. Men that did not have control over their partner were seen as lacking “dignity and respect” [91]. Hence, a study in Ecuador reported that male RJ followed by physical IPV was considered commonplace and expected [83], as it functioned as a mechanism to reassert male control and authority.

Women who refused sex also reported facing suspicions of infidelity followed by physical and psychological IPV [88,91]. For example, a man in South Africa reported his girlfriend had come to his house in the evening after drinking alcohol, but did not want to have sex, implying to him that she had already had sex with someone else. He stated he had been “humiliated” and “had to lay a hand on her [hit her] because of what she did” [91].

A study from Ghana suggested that suspicions of infidelity could also lead to femicide, and the subsequent suicide of the male partner [79]. In seven cases of femicide–suicide extracted from a major newspaper, it was found to be most often precipitated by women being out of the house (or asking permission to leave) and their husbands suspecting them of being interested in other men. It was also sparked by women engaging in phone calls and text messages, and in cases of pregnancy where the partner suspected it was a result of infidelity [79].

Evidence from across the world indicated that women who were suspected of infidelity also experienced psychological IPV, including being insulted [88,93,100], threatened [89,92] and ignored [96,102] by their partners. Psychological IPV often occurred in conjunction with physical IPV and controlling behaviours (Pathway 3) [84,92,93,100,102].

**Pathway** **2.** Women who suspect their partner of infidelity experience physical and psychological IPV.

In some studies, men reported facing pressure from their peers to have sex with many women to affirm their masculinities; men who did not have multiple partners were reportedly described as “controlled by their wives” or “cowards” [84,91]. Hence, women asking their partners to be faithful or accusing them of infidelity constituted a direct threat to their aspired masculinities, which was interpreted to have implications for the IPV that followed.

Participants reported that male alcohol consumption, phone calls and community gossip triggered female RJ [100,103], and that women confronting their partners about infidelity led to arguments about whether the infidelity had occurred [92,99], which in turn escalated to physical and psychological IPV [80,87,88,92,95,100,102,103].

Women in a study in Nicaragua reported that they, therefore, stopped asking their partner to be faithful because it would bring conflict, while men reported that they would not listen to their partner if they did ask them to be faithful [84]. Confrontations about male infidelity could also lead to men suspecting their wives of infidelity in return (Pathway 1): As the author of one study reported:

“Most [participants] said that their husband became suspicious when they (the wife) questioned them concerning their [sexual] behaviors while drinking. This suspicion frequently resulted in the husband physically or verbally abusing them. The women said that their husbands often assumed such questions reflected the wife’s own infidelity, which further angered the husbands”

[103] (p. 818)

Reports of bidirectional IPV (male-to-female and female-to-male) triggered by infidelity or RJ also emerged [87,89,90,95,100,102]. These confrontations usually included alcohol consumption, and increasingly severe physical IPV against women [87,89,102], sometimes resulting in femicide [90].

Participants noted that when men drank alcohol and used drugs, women became more suspicious of infidelity, and men became more violent, leading to increased IPV [80,85,87,90,95,99,102,103], and likewise when men decreased their consumption, suspicions of male infidelity and male violence decreased [87].

In the USA there was also evidence of women using accusations of infidelity and homosexuality to threaten their partners’ heteronormativity and attainment of hegemonic masculinities, leading to IPV. As a man said:

“That’s what really made me snap when you said, ‘go fuck [male friend], go fuck [male friend], go back to your boyfriend…” That really hurt my whole manhood, my dignity, my pride, my everything”

[95] (p. 945)

Findings of bidirectional IPV came primarily from the USA and Europe—contexts with more equal gender dynamics, and where women are less likely economically dependent on their partners—which may partially explain higher rates of bidirectional violence [89,90,95,102].

These findings about bidirectional IPV triangulate quantitative results that the association between mutual RJ and IPV is greater among reciprocally violent couples. It also provides some support for the explanation put forth by authors of the included quantitative studies citing a “lack of emotional regulation and conflict resolution skills” among intimate partners as the reason for the association between infidelity, RJ and IPV, which also arose primarily from those studying populations in the USA. Additional support for this theory emerged from quantitative findings that intimate partner femicides committed with excessive injury were more common when RJ was the motive.

#### 3.9.2. Mechanism B: Accusations of Female Infidelity Are Associated with Threatened Femininities and Violence

**Pathway** **3.** Men who anticipate partner infidelity use controlling behaviours and economic IPV.

A widespread finding was that men often used controlling behaviours to limit woman’s autonomy and prevent infidelity. Participants reported that men controlled their partners by destroying their property [84], monitoring and restricting their access to text messages, calls and social media [84,91,96,101], frequently calling them to monitor their movements [91,96,100], isolating them from friends and family [84,92,96,100] and in extreme cases, not allowing them to leave the house [92,96]. As a midwife described:

“This man used to keep his wife inside the house, when he goes to work, he locks the door and sweeps the garden neatly, so that [he will know] if someone comes to the house [because] they will leave foot prints [in the sand]”

[92] (p. 7).

Underlying these behaviours—according to participants—were men’s fear of losing their partner, a lack of trust in their partner and a perception that women were easily persuaded into infidelity [92,94,96,101]. As a man explained:

“As you know women admire a lot and so at work is where she might find someone to admire her and then change her mind into cheating”

[94] (p. 5)

Evidence of economic IPV also emerged, where women who were repeatedly accused of infidelity reported their husbands threatened to “chase them” from home [92,98]. A woman in Indonesia also described how her husband spread rumours about a supposed infidelity to hurt her educational and career development prospects:

“He directly came to the school principal and raised negative speculations [accused her of infidelity] that ruined my reputation. He said, “if something bad happened in the future at this school, don’t blame me as I’ve already warned you about her.” So the next day the principal called me and cancelled my promotion for the master program”

[98] (p. 6)

This example highlights how the social stigma associated with female infidelity in a patriarchal context gave men and institutions power and leverage over women; they could use accusations of infidelity to justify IPV, and had the ability to leave them destitute, or to hinder their ability to get an education and join the workforce.

Studies from developing countries found that as countries modernized, and gender norms shifted—with women becoming more empowered—these triggers could be exacerbated [82,92,94,98]. Women gaining employment and spending more time outside of the home led to increased male accusations of infidelity [82,84,97]. Similar social tensions were described by a Romanian immigrant in Spain, where traditional values from the country of origin clashed with the modern lifestyle women were leading in the host country, leading to increased community gossip, male control and violence (Pathway 1) as women tried to navigate dressing in modern clothing, going to bars and interacting with people—especially men—outside of the immigrant community [97].

In a study conducted in Nicaragua, male and female participants reported that when control was exerted in the name of RJ, it was often interpreted as an expression of love and the behaviour was tolerated [84]. As a man explained:

“If I’m not interested in her, I don’t care what she does. If she goes out with some person, she can do what she wants with her life. But if I had feelings for her, it will affect [hurt] me”

[84] (p. 627)

It was not always clear, however, to what extent and in which contexts controlling behaviours were used because of a real anticipation of infidelity, or as an excuse to maintain hegemonic masculinities (Mechanism A). In the post-war context of Sri Lanka, for example, a study found that increases in female employment coincided with decreases in men’s employment opportunities, leading to shifting power dynamics, and tensions in the relationship. Men who had been replaced by their wives as the main provider for the family tried to reassert control through monitoring their wife’s movements and through physical violence [92].

**Pathway** **4.** Women experience accusations of infidelity as a form of psychological IPV.

Accusations of infidelity reportedly caused female participants great emotional distress [88,92,98,102]. Hegemonic femininities depict women as passive recipients of sex, with few sexual needs, whose worth decreases as their sexual experience increases [104]. Unfaithful women are often seen to have broken these gendered norms, by assertively seeking sex outside their marriage for their own sexual gratification. Hence, accusations of infidelity represent a direct threat to hegemonic femininities. It was not clear in the data, however, if men intentionally used mechanisms of threatened femininities to inflict violence.

Sometimes triggers such as HIV or pregnancy—in which paternity doubts triggered male RJ—were found to precipitate accusations of infidelity experienced as psychological IPV [93,100]. For example, a pregnant woman in South Africa described her partner calling her a “slut” after testing positive for HIV [93], while a woman in Brazil described her partner’s reaction to an unplanned pregnancy:

“Lots of swearing at me, abusing me, saying that the child was not his… After we had the child it became worse… So all the agony began, the bad relationship, him complaining about everything, saying that I should’ve taken care of myself (the pregnancy) [had an abortion]”

[100] (p. 1044)

Disrupting gender norms could also trigger accusations of infidelity experienced as psychological IPV. For example, in Bangladesh where it is customary for men to eat before their wives, a woman explained:

“On a few occasions I would be so hungry that I ate my meal before. That made him so angry that he berated me and said, ‘You probably have another husband whom you fed first and that’s why you have had your food before me.’”

[88] (p. 113)

In these examples it is unclear how much the accusations of infidelity were spurred by a real suspicion of infidelity or actual infidelity (Pathway 1), versus as an intention to hurt the recipient.

#### 3.9.3. Mechanism C: Beliefs about Infidelity and Sex Are Associated with Patriarchal Culture and Sexual Violence

**Pathway** **5.** Women who anticipate partner infidelity and male suspicion of their own infidelity experience sexual coercion.

A gendered double standard around infidelity emerged. In some studies male infidelity was seen as “normalized” and something that “has to be” [84,91], while in South Africa participants did not believe that women had the same biological imperative as men to have many partners [91], and in Nicaragua female infidelity was seen to be “like death”, and unfaithful women faced severe stigma and discrimination [84].

Studies found that both men [82,91] and women [84,103] often perceived sex as a “basic right” for men and marital duty for women. Past research suggests that male sexual entitlement is supported by patriarchal beliefs that men’s desires take precedent over those of women [105]. The counterpoint is the belief that is it a women’s duty to fulfil men’s sexual needs. This belief has been found to be particularly strong in intimate partnerships, where gender norms dictate that women should acquiesce to sex [106].

Relatedly, we found that men sometimes believed that when women did not want to have sex, it was a sign that they had been unfaithful [84,88,91,103]. A man in Nicaragua noted that these beliefs were perpetuated in soap operas, where he learned that if “you want it [sex] and she doesn’t, [she says] I’m tired” it is a clear indication that she “has a lover” [84]. Some women, therefore, reported “overcompensating sexually” by never refusing sex—even when they would have liked to—to avoid making their partner suspicious [84]. In India participants noted that men’s suspicion of infidelity was long-lasting and “one will have to repent for life” if he begins to suspect you [103].

In India and Nicaragua women reported fear that if they did not have sex with their husband, then he would seek sex with other women, so they engaged in sex even when they did not want to [82,84,103]. A belief reinforced many times by other women in the family, as one participant described:

“My sister-in-law and my mother also told me that men go to other women when his wife did not satisfy him [sexually]. So when he wants to do [have sex] I make myself ready”

[82] (p. 132)

Some women blamed themselves for their partner’s infidelity [80,81,84], demonstrating an internalization of patriarchal norms. In some studies, this was compounded by societal beliefs that men are unfaithful because women fail to meet their sexual needs at home, or are not ‘desirable’ enough to their husbands, forcing them to look for a sexual partner outside the marital relationship [78,80,84]. A woman explained:

“I felt dishonored when others knew my husband had engaged in an affair with another woman. Some friends looked down on me and said I could not control my husband. It looked like I could not complete the roles of a wife… some gossiped and told me… I might have something wrong with me so he couldn’t stand me... It is my karma in my past life that made me live with my husband in this life”

[80] (p. 352)

This quote exemplifies the stigma and discrimination many women faced because of their partner’s infidelity due to patriarchal beliefs about male entitlement to sex, and the hopeless tone women took when discussing it.

**Pathway** **6.** Women who suspect male infidelity are unable to negotiate condom use.

Having sex without a condom was seen as a sign of love and trust in some studies [84,91], and in Nicaragua women reported that despite fear that their partners had contracted HIV or other STIs through extramarital affairs, they were unable to negotiate condom use as this would be interpreted as lack of trust in the relationship [84]. Studies in India and South Africa also found that asking a partner to use a condom was tantamount to accusing them of infidelity, or admitting to it yourself [91,103].

In studies from India and Puerto Rico women reported that they had no power to negotiate condom use in their relationships, but underlying this lack of control was an inability to voice their suspicions of infidelity for fear of physical violence (Pathway 2) [78,103]. Evidence that infidelity, HIV and physical violence were intertwined also emerged in sub-Saharan Africa, and in one instance a woman was reportedly murdered after rebuffing her husband’s sexual advances and telling him she feared he had been unfaithful and contracted HIV [79,87].

Other studies found that women who did report asking their partners to use condoms were met with refusal [78,84], mockery [84] and threatened abandonment [78]. In Nicaragua, while three women reported successfully convincing their partners to use condoms after their infidelity had been exposed, for two of them the condom use only lasted two weeks [84].

## 4. Discussion and Implications for Research and Programs

This is the first review—to our knowledge—that systematically examines the evidence on the association between real or suspected infidelity and RJ, and IPV against women. Across quantitative and qualitative studies conducted in all regions, we found that real or suspected infidelity and RJ were strongly related to IPV, which merits significant investment in the field of IPV prevention. Three mechanisms emerged from the data: (1) hegemonic masculinities, which when threatened can lead to physical and psychological violence; (2) hegemonic femininities, which are a powerful tool for controlling behaviours and inflicting economic and psychological IPV and 3) patriarchal beliefs about infidelity and sex, which can be used to justify sexual IPV; making up a total of six pathways (see Figure 2). 

The pathways describe how both male and female suspected infidelity and anticipated infidelity, as well as anticipated partner suspicions of female infidelity and accusations of female infidelity, can all lead to different forms of IPV against women. Notably, we found evidence that women experience the pathways in an iterative way—moving fluidly within and between them—which is important when considering how they represent lived experiences of IPV. These pathways provide an unprecedented opportunity to unpack the association between infidelity, RJ and IPV, with important implications and applications for programming and research. Additionally, we found a lack of communication skills, economic control and dependency, and alcohol interacted with these pathways to increase risk of IPV. Finally, our study results highlight several gaps in the literature base, including the need to increase internal and external validity of quantitative studies, and transferability of qualitative findings.

### 4.1. Theoretical Implications

Our synthesis of the literature provides critical evidence for the theories underpinning research on infidelity, RJ and IPV. The “patriarchal culture” and “threatened masculinities” explanations put forth by authors of included quantitative studies were strongly supported by our identified mechanisms and pathways derived from qualitative studies, while the “lack of emotional regulation and conflict resolution skills” explanation received some support in the context of bidirectional IPV in the USA and Europe (see Figure 2). We did not find any qualitative evidence to support the “evolutionary or biological explanation” put forth by other authors.

The patriarchal context is responsible for the creation of hegemonic masculinities and femininities [107], and we found that when these masculinities were threatened by women suspecting their partner of infidelity, gaining employment or by men becoming suspicious of their partner’s infidelity, then this could result in RJ and violence (Mechanism A). Additionally, and in line with global evidence (e.g., [108]), we found that unlike unfaithful men, unfaithful women faced severe stigma and discrimination. Hence, patriarchal, cultural expectations that women will be subservient and faithful to their partners gives men leverage over them, indicating that hegemonic femininities—which were threatened by accusations of infidelity—were missing from the explanations proposed by the authors of included quantitative studies (Mechanism B) [109].

We also found that these patriarchal norms, along with beliefs that men are entitled to sex and it is a women’s role to fulfil those sexual desires, acted as mechanisms for sexual violence (Mechanism C). Additionally, condom use appeared to be inconsistent with ideas of fidelity, “real love” and trust in intimate relationships, an observation that is well documented in the literature (e.g., [110]). Finally, we found some evidence of paternity doubts leading to male RJ and IPV, and more research is needed to determine whether reproductive coercion through contraception control could be an additional pathway between infidelity, RJ and IPV [111].

### 4.2. Programing Implications

There has been heavy investment in the field testing strategies to target structural patriarchal norms and institutions [112,113], and challenge harmful masculinities (e.g., seeking multiple sexual partners to assert status and men as financial providers) and femininities (e.g., women acquiescing to sex in partnerships and internalizing patriarchal tenets), emphasizing alternative gendered identities and relationships that are grounded in equality and respect [114]. This work to change social norms is foundational to decreasing tolerance for IPV and increasing social sanctions for perpetrators [115]. Much of this gender-transformative work has happened in either workshops (not designed for couples) or through community mobilization campaigns. These aim to not only change individual behaviours but to create social environments supportive of non-violent relationships, equalizing norms regarding faithfulness and infidelity, and lessening the social currency of suspected infidelity or RJ as justifiable reasons to engage in violence, with some documenting reductions in IPV in randomized control trials [113].

Missing from much of this work, are couples-based interventions, an understudied component of IPV-related work. While couples-based work has demonstrated success for HIV-prevention and treatment programming [116], this has been a less popular strategy in IPV-prevention work. This work has historically been challenging to enact as the theoretical underpinnings can clash with traditional feminist theories on the root causes of violence and why and how men perpetrate violence, as well as the safety, legal and ethical considerations of engaging with men who may be actively perpetrating violence [117]. A skills-based, communications and conflict-resolution programming for couples, delivered through a gender equitable framework that specifically disrupts traditional patterns of infidelity and RJ, could be a powerful violence prevention strategy. This has been best exemplified by the Indashyikirwa intervention in Rwanda, which successfully halved reports of physical and sexual IPV through a couples curriculum, community mobilization, training and engagement of opinion leaders and women’s safe spaces [118].

Qualitative work conducted in rural South Africa in the context of gender-transformative programming, not designed for couples, found that for many participants who wanted to enact more gender equitable and non-violent behaviours, it was difficult to consistently act upon those new values, especially for those with a previous history of either experiencing or perpetrating violence [119]. As the field continues exploring this strategy, program developers should work directly with the couples they wish to impact—incorporating their perspectives during development—and creating interventions that aim to increase relationship quality [23,106]; especially by promoting open communication around sex, monogamy, trust and STIs. Alcohol has also been established as an important trigger for IPV, both in our findings and in the wider literature [120], and couples-based substance use programs have had documented success in reducing alcohol and substance use, and IPV [121], and merit consideration for inclusion in couples-based IPV prevention programming. Additionally, our findings highlight the need to address harmful norms that RJ or having sex without a condom are a sign of love and trust in a relationship, or that women should not work outside of the home.

Female economic empowerment has been an increasingly popular IPV prevention strategy too, as economic dependence has been identified as a major risk factor for all types of IPV [122]. Our results suggest, as has been seen in other settings, the need to simultaneously address female economic dependency and patriarchal gender norms. In line with past research, we found that women gaining employment outside of the home increased accusations of infidelity, controlling behaviour and violence [123]. This was seen in low-and middle-income countries (as well as in an immigrant community in Spain); contexts in which women do not traditionally work outside of the home [124], and attitudes that promote male control are prevalent. Hegemonic masculinities tied to being the primary financial provider underlie this increase in control following female employment [125]. We found that women earning money could threaten these dominant social and gender norms, leading to IPV intended to reassert the gendered hierarchy, and supporting the aforementioned “threatened masculinities” explanation. In Rwanda an alternative explanation was identified, where women bringing home money triggered male suspicion of infidelity because men assumed that his partner had not earned the money but had been given it from another man (a sign of infidelity) [126]. These findings come primarily from qualitative studies, as we did not find this level of nuance in quantitative papers, and more mixed-methods research is needed to fill this gap.

Programs and policies aimed at increasing women’s economic independence and empowerment must reckon with the deep interplay between economics and gender norms. Cash transfer projects have shown success across settings in reducing IPV [127]; with qualitative research in some settings finding that part of that success was due to the program operating within acceptable gender norms (i.e., providing women money to spend on food for their children) [24]. Microfinance and entrepreneurship programs, however, have a weaker empirical literature base to date, with studies finding mixed results on IPV outcomes (e.g., [128]), potentially due to the increased stressors of taking on financial risks inherent in running a business, or women spending less time in the home. Explicitly addressing the role of perceived infidelity and RJ in the context of economic empowerment work may be an important factor to buoy the effectiveness of such programs that shift the economic power dynamic in a family or facilitate women engaging in the labour force. Another potential mechanism of decreased violence, in addition to the aforementioned reduction in threatened masculinities, includes a reduction in poverty-related stress and conflict within the couple, decreasing miscommunications—including those about infidelity and RJ—leading to IPV [24,94,127].

### 4.3. Gaps in the Literature

Our review highlights important gaps in the literature. Women were disproportionately sampled, with little evidence available on men’s experiences of RJ and real or suspected partner infidelity leading to IPV, and this should be explored further. Additionally, few included studies measured economic IPV, although this was an important qualitative theme, and economic IPV has been found to have an independent negative effect on women’s health, beyond other types of IPV [5]. Furthermore, the included studies were generally methodologically weak, and ranked poorly on our quality assessment. None of the included studies were population-based, and instead over relied on convenience sampling; and only a limited number provided longitudinal data on RJ or infidelity and IPV, hindering our ability to clearly establish causality. Included studies were also disproportionately representative of Western countries—especially the USA—where more research has been conducted. This is partially due to data from US studies being more likely to be disaggregated for participants aged 18 years and over, whereas studies conducted in sub-Saharan Africa, for example, often combined findings on participants aged 15 years and over and were thus excluded.

The available evidence documented in the review suggests substantial variation in the frequency with which RJ or infidelity was cited as a reason for IPV. This may be due in part to differing cultural attitudes towards RJ and infidelity [129], and IPV [130]. More research is needed to determine whether the mechanisms and pathways identified in this review are upheld across different regions (e.g., the Middle East and North Africa) and countries, as well as among different populations within countries.

### 4.4. Measurement Implications

Heterogeneity of measurement outcomes and tools may also provide an explanation for the wide range of quantitative findings reported. Only approximately one-third of reviewed studies used a validated questionnaire or scale to measure infidelity or RJ. Historically, RJ has been subsumed into bigger psychological aggression variables—such as in the multidimensional measure of emotional abuse restrictive engulfment subscale [131], which was designed to pick up on jealous behaviours such as asking where your partner had been and who they had been with in a “suspicious manner”—or conflated with forms of IPV such as the subscale on controlling behaviours in the CTS2 [75]—which includes items such as “acts suspicious and jealous of the other one”. The lack of conceptual clarity on what constitutes RJ likely plays a role in the limited research that has been conducted on the topic. Hence, research on RJ should employ a standardized, validated measurement tool to ensure high-quality results and allow for future meta-analyses, while distinguishing RJ from larger measures of psychological IPV or controlling behaviours. Additionally, researchers could use the pathways identified in this review to develop research questions, as well as items for a standardized and comprehensive scale for measuring RJ in IPV studies.

Our findings also support distinctly measuring accusations of infidelity, anticipated infidelity, suspicions of infidelity, confirmed infidelity and RJ, as they lead to IPV along differing pathways. Accusations of infidelity can be experienced as psychological IPV, but these accusations do not always stem from RJ, and they may sometimes be used intentionally to hurt their partner, or as a mechanism to justify controlling behaviours and violence. We also found that infidelity and RJ are often distinct from IPV and precede it on a pathway to violence. Measures of infidelity should be precise, and clearly indicate which partner has been unfaithful and the degree of the respondent’s certainty, as these can operate along different pathways to violence.

### 4.5. Limitations

Our review was limited to studies published within the last 10 years, the aim of which was to yield recent findings relevant to ongoing and future research and prevention efforts. With the intent of only including rigorously evaluated studies, we also limited our search to peer-reviewed publications. We did not conduct a forward or backward reference search of our included studies given the large number of studies included and the strong search strategy implemented. However, a particular strength of this study is our inclusion of quantitative and qualitative data, which allowed us to make meaningful programming and research recommendations. We also followed methods used in previous peer-reviewed publications to ensure analytical rigor by only reporting on trends supported by abundant evidence that emerged across multiple studies [132] and drawing our results more heavily from high-quality studies [133,134].

To our knowledge, this review is the first to comprehensively analyse the peer-reviewed evidence from the past 10-years on infidelity, RJ and IPV, making an important contribution to the field. Our results highlight opportunities to improve research and IPV prevention efforts, in particular around the relational level of the ecological model, which has been historically understudied.

## 5. Conclusions

Fifty-one papers from 28 countries were included in our review and the evidence showed a consistent association between real or suspected infidelity or RJ and IPV against women. Three mechanisms—with two pathways within each mechanism—emerged from the data, for a total of six pathways that further elucidate this association (see Figure 2). As outlined above, this study highlights opportunities to improve research—including the implementation of standardized measurement tools that make the distinction between RJ and suspected, confirmed and accusations of infidelity—and IPV prevention efforts, including gender-transformative programming that accounts for infidelity and RJ, and couples interventions that focus on improved communication and trust. Infidelity and RJ should be prioritized in future IPV research and programming, shedding light on these historically understudied relational level drivers of violence against women.

## Figures and Tables

**Table 1:** Inclusion and exclusion criteria and rationale.

Criteria	Included	Excluded	Rationale
Sampling	Adults (aged 18+) in current heterosexual relationships.	Students, people with diagnosed medical conditions.	Association between infidelity and RJ, and IPV may differ in: - adolescent and student relationships because of school-level norms (e.g., non-exclusivity in relationships, social acceptance of physical and sexual violence) [32,33], the importance of same-sex friendships [32,34] and frequent use of social media (which has been found to act as a trigger for RJ and a mechanism for monitoring behaviour) [35]. - relationships that are ending or have ended, as the triggers of RJ and IPV during the ‘process of leaving’ and after leaving the relationship seem to be unique [36]. For instance, the threat of losing a partner has been found to increase the frequency and severity of IPV, as well as the risk of femicide [37,38], suggesting this is different from ‘typical’ patterns of IPV in on-going relationships. Additionally, violence by a former partner has been found to be greater when RJ is triggered by the woman having a new partner [38], and is associated with different experiences of IPV including intrusiveness and stalking [39,40]. - people diagnosed with medical conditions as mentally ill populations (e.g., schizophrenia, bipolar disorder and anxiety disorder) have been found to be more likely to perpetrate IPV than healthy populations, suggesting different triggers [41]. The triggers of RJ in pathological jealousy are also likely to differ as they may not be rooted in reality. Pathological jealousy is distinguished from normal RJ by obsession and delusion, including severe and irrational jealous thoughts, feelings and behaviours, and the inability to change these when confronted with contradicting evidence [42].
Infidelity or romantic jealousy (RJ) outcome	Quantitative: Prevalence of, or measure of association between, infidelity or RJ and male-to-female IPV.	Infidelity or RJ as part of bigger variable without disaggregates, proxy’s such as “polygamy”.	- proxy’s that don’t specifically refer to infidelity or RJ, as they may suggest different cultural contexts or situation in which sexual relations with someone outside of the relationship were experienced differently (e.g., open relationships). - female-to-male IPV and IPV in same sex relationships because of ubiquitous patriarchal norms that create unequal power dynamics within the couple [26]. Some evidence also suggests that women perpetrators of IPV may be more motivated by retaliation, while men may be more motivated by control [43], and that same-sex relationships are more likely to be consensually non-monogamous [44].
Intimate partner violence (IPV) outcome	Qualitative: Evidence of pathway between infidelity or RJ and male-to-female IPV.	Predicted or feared IPV.	

**Table 2:** Country, sample size and quality assessment of included quantitative studies.

Author (Year)	Country	Sample Size	Quality ^1^	Reference
Alan et al. (2016a)	Turkey	1039	★☆☆☆	[49]
Alan et al. (2016b)	Turkey	442	★☆☆☆	[50]
Ansara and Hindin (2009)	Philippines	1861	★★★★	[51]
Chuemchit et al. (2018)	Thailand	2462	★★★☆	[52]
Conroy (2014)	Malawi	422	★★★☆	[53]
Edelstein (2018)	Israel	194	★★★☆	[54]
Goetz and Shackelford (2009)	USA	546	★★☆☆	[55]
Graham-Kevan and Archer (2011)	UK	43	★☆☆☆	[56]
Guay et al. (2016)	Canada	466	★★☆☆	[57]
Kalokhe et al. (2018)	India	100	★★☆☆	[58]
Kerr and Capaldi (2011)	USA	153	★★★☆	[59]
LaMotte et al. (2018)	USA	589	★★★☆	[60]
Madsen et al. (2012)	USA	258	★★☆☆	[61]
McKay et al. (2018)	USA	1332	★★☆☆	[62]
Messing et al. (2014)	USA	432	★★☆☆	[63]
Paat et al. (2017)	USA	5000	★★★☆	[64]
Salwen and O’Leary (2013)	USA	830	★★★★	[65]
Shrestha et al. (2016)	Nepal	404	★★☆☆	[66]
Snead et al. (2019)	USA	318	★★☆☆	[67]
Stieglitz et al. (2011)	Bolivia	49	★★☆☆	[68]
Stieglitz et al. (2012)	Bolivia	266 ^2^	★☆☆☆	[69]
Toprak and Ersoy (2017)	Turkey	162	★★☆☆	[70]
Townsend et al. (2011)	South Africa	428	★☆☆☆	[71]
Ulibarri et al. (2010)	Mexico	300	★☆☆☆	[72]
Wang et al. (2009)	China	2661	★★☆☆	[73]

^1^ Studies were quality assessed on 8 core criteria that evaluated external and internal validity, scoring 0 or 1 for each, for a total possible score of 8. Studies scoring 7–8 points received ★★★★, studies scoring 5–6 points received ★★★☆, studies scoring 3–4 points received ★★☆☆ and studies scoring 2 points and lower received ★☆☆☆. ^2^ Sample size varied by question due to missing data.

**Table 3:** Characteristics and findings of included quantitative studies (*n* = 25).

Characteristics and Findings	No. Studies (%)	Studies
**Region** ^1^		
East Asia and Pacific	3 (12)	[51,52,73]
Europe and Central Asia	4 (16)	[49,50,56,70]
Latin America and Caribbean	3 (12)	[68,69,72]
Middle East and North Africa	1 (4)	[54]
North America	10 (40)	[55,57,59,60,61,62,63,64,65,67]
South Asia	2 (8)	[58,66]
Sub-Saharan Africa	2 (8)	[53,71]
**Study Design**		
Cross-sectional	22 (88)	[49,50,51,52,53,54,55,56,57,59,60,61,63,65,66,67,68,69,70,71,72,73]
Longitudinal-Cohort	3 (12)	[58,62,64]
**Infidelity or RJ Measurement Instrument**		
Validated questionnaire or scale	8 (32)	[52,57,58,60,61,62,65,67]
Continuous or Likert scale question	4 (16)	[53,55,56,68]
Multiple choice or binary question	7 (28)	[51,63,64,66,71,72,73]
Open-ended question	3 (12)	[49,50,69]
Observational	2 (8)	[54,70]
Mix	1 (4)	[59]
**Infidelity or RJ Outcome** *		
F suspicion of M infidelity	4	[53,62,64,66]
M suspicion of F infidelity	5	[52,53,55,62,71]
Real F infidelity	2	[53,55]
Real M infidelity	7	[53,55,58,66,68,71,72]
F romantic jealousy	6	[57,60,61,62,65,73]
M romantic jealousy	12	[51,52,56,57,58,60,61,62,63,65,67,73]
Romantic jealousy not specified	1	[59]
All (open-ended/observational)	5	[49,50,54,69,70]
**IPV Measurement Instrument**		
Conflict Tactics Scale or adaption	9 (36)	[51,56,57,60,61,62,63,65,67]
Other scale (e.g., Sexual Coercion in Intimate Relationships Scale)	4 (16)	[55,58,64,72]
Inventory of specific behaviours (e.g., pushed or shoved)	6 (24)	[49,50,52,66,71,73]
General items (e.g., experience of “violence” or “assault”)	4 (16)	[53,59,68,69]
Intimate partner homicide	2 (8)	[54,70]
**IPV Outcome**		
Physical only	8 (32)	[54,56,59,64,68,69,70,73]
Sexual only	3 (12)	[55,65,67]
Psychological only	0	
Economic only	0	
Physical or sexual	3 (12)	[53,62,71]
Physical or psychological	3 (12)	[57,60,61]
Physical, sexual, or psychological	6 (24)	[51,52,58,63,66,72]
Physical, sexual, psychological or economic	2 (8)	[49,50]
**IPV Outcome Reporter**		
Female self-report	15 (60)	[49,50,51,52,56,59,61,62,63,64,66,67,68,69,72]
Male self-report	3 (12)	[58,60,71]
Couple report	5 (20)	[53,55,57,65,73]
Observation (e.g., review of court data)	2 (8)	[54,70]
**Analysis Type** *		
Prevalence (univariate)	12	[49,50,51,54,56,57,60,61,66,67,70,72]
Unadjusted or bivariate	10	[52,55,56,57,58,60,61,65,66,72]
Adjusted or multivariate	14	[51,53,58,59,62,63,64,66,67,68,69,71,72,73]
**Infidelity or RJ and IPV Association** *		
Infidelity or RJ decreased IPV	0	
Infidelity or RJ increased IPV	19	[51,52,53,55,56,58,59,60,62,63,64,65,66,67,68,69,71,72,73]
Not associated	5	[53,62,63,64,71]
**Mechanisms Described by Authors to Explain Findings** *		
Evolutionary or biological	5	[55,56,67,68,69]
Lack of emotional regulation and conflict resolution skills	7	[57,59,60,61,62,65,73]
Patriarchal culture	9	[49,50,55,60,64,66,70,71,72]
Threatened masculinities	4	[53,54,58,62]
None given	3	[51,52,63]

1 Classified by World Bank regions [74]. * Count >25 as some studies provide multiple.

**Table 4:** Characteristics of included qualitative studies.

Author (Year)	Country	Sample Size	Type of IPV	Quality ^1^	Reference
Abreu et al. (2010)	Puerto Rico	39	Physical, sexual	★★★☆	[78]
Adinkrah (2014)	Ghana	35	Physical (femicide)	★★☆☆	[79]
Arpanantikul (2010)	Thailand	18	Physical, psychological, economic	★★★★	[80]
Bahadir-Yilmaz and Oz (2019)	Turkey	30	Physical, sexual	★☆☆☆	[81]
Berg et al. (2010)	India	44	Physical, psychological, economic	★★★☆	[82]
Boira et al. (2017)	Ecuador	61	Physical	★☆☆☆	[83]
Boyce et al. (2016)	Nicaragua	30	Physical, sexual, psychological	★★★☆	[84]
Byun (2012)	USA	~95 ^2^	Physical	★☆☆☆	[85]
Conroy et al. (2018) ^†^	Malawi	50	Physical, economic	★★★☆	[86]
Conroy et al. (2019) ^†^	Malawi	50	Physical	★★★☆	[87]
Das et al. (2016)	Bangladesh	42	Physical, sexual, psychological	★★☆☆	[88]
Fenton and Rathus (2009)	USA	24	Physical, psychological	★★☆☆	[89]
Freysteinsdóttir (2017)	Iceland	11	Physical (femicide)	★★★☆	[90]
Gibbs et al. (2014)	South Africa	~63 ^3^	Physical	★★★☆	[91]
Guruge et al. (2017)	Sri Lanka	30 ^4^	Physical, psychological, economic	★★★☆	[92]
Hatcher et al. (2016)	South Africa	32	Physical, psychological	★★★★	[93]
Kyegombe et al. (2014)	Uganda	40	Economic	★★★★	[94]
Nemeth et al. (2012)	USA	34	Physical	★★★☆	[95]
Nhi et al. (2018)	Vietnam	20	Psychological	★★★★	[96]
Nudelman et al. (2017)	Georgia, Romania, Spain ^5^	10 ^6^	Physical	★★★☆	[97]
Nur Hayati et al. (2013)	Indonesia	7	Physical, psychological, economic	★★★☆	[98]
Orengo-Aguayo and Lawrence (2014)	USA	40	Physical, psychological	★★★★	[99]
Paixão et al. (2014)	Brazil	19	Physical, psychological	★★☆☆	[100]
Starmann et al. (2017)	Uganda	20	Sexual, psychological	★★★☆	[101]
Stith et al. (2011)	USA	22	Physical, psychological	★★★☆	[102]
Varma et al. (2010)	India	14	Physical, sexual, psychological	★★★☆	[103]

^†^ Papers with mating notation report on data from the same study. ^1^ Studies were quality assessed on 10 core criteria that evaluated validity, ethics, data analysis, presentation of findings and the value of the research. Studies were scored on a scale of 0–2, for a total possible score of 20. Studies scoring 18–20 points received ★★★★, studies scoring 15–17 points received ★★★☆, studies scoring 12–14 points received ★★☆☆ and studies scoring 11 points and lower received ★☆☆☆. ^2^ Ninety-five anonymous online posts were analysed; some may have come from the same poster. ^3^ Nineteen men were randomly selected from all who enrolled in the intervention to participate in interviews and could have overlapped with the 44 men who participated in focus group discussions. ^4^ Fifteen women who had experienced IPV and 15 service providers who were knowledgeable about IPV. ^5^ In Spain 2 immigrants from Latin America and 1 from Romania were interviewed. The other Romanian woman who was interviewed had immigrated to Italy and then returned to Romania. ^6^ Six in Georgia, 1 in Romania and 3 in Spain.

## Appendix A

Detailed search strategy adapted from search terms used in reviews on IPV [28,29] and infidelity and romantic jealousy [15,30,31].

**Table A1:** Detailed search strategy.

Database	Search Script for Intimate Partner Violence	Search Script for Infidelity and Romantic Jealousy
ASSIA (Applied Social Sciences Index and Abstracts)	MAINSUBJECT.EXACT.EXPLODE(“Domestic violence”) OR MAINSUBJECT.EXACT.EXPLODE(“Battered women”) OR ((DE=”domestic violence”) or (DE=“battered women”) or (abuse* within 3 wom*n) or (abuse* within 3 spous*) or (abuse* within 3 partner*) or ((wife within 3 abuse*) or (wives within3 abuse*)) or ((wife within 3 batter*) or (wives within 3 batter*)) or (partner* within 3 violen*) or (spous* within 3 violen*))	MAINSUBJECT.EXACT.EXPLODE(“Jealousy”) OR MAINSUBJECT.EXACT.EXPLODE(“Marital infidelity”) OR ((jealous*) or (infidelit*) or (“sexual affair*”) or (unfaithful*) or (adultery) or (adulterer) or (extramarital) or (“controlling behaviour*”) or (“controlling behavior*”))
CENTRAL (Cochrane Central Register of Controlled Trials)	#1 (battered wom*n): ti #2 (battered wom*n): ab #3 MeSH descriptor: [Battered Women] explode all trees #4 MeSH descriptor: [Domestic Violence] this term only #5 MeSH descriptor: [Gender-Based Violence] this term only #6 MeSH descriptor: [Intimate Partner Violence] explode all trees #7 MeSH descriptor: [Spouse Abuse] explode all trees #8 abuse near/3 (woman or women): ti #9 abuse near/3 (woman or women): ab #10 abuse* near/3 partner*: ti #11 abuse* near/3 partner*: ab #12 abuse* near/3 spouse*: ti #13 abuse* near/3 spouse*: ab #14 wife near/3 batter* or wives near/3 batter*: ti #15 wife near/3 batter* or wives near/3 batter*: ab #16 wife* near/3 abuse* or wives near/3 abuse*: ti #17 wife* near/3 abuse* or wives near/3 abuse*: ab #18 violen* near/3 partner* or violen* near/3 spous*: ti #19 violen* near/3 partner* or violen* near/3 spous*: ab #20 violen* near/3 date or violen* near/3 dating:ti #21 violen* near/3 date or violen* near/3 dating:ab #22 #1or#2or#3or#4or#5or#6or#7or#8or#9or#10or#11or#12or#13or#14or#15or#16or#17or#18 or#19or#20or#21	#23 MeSH descriptor: [Jealousy] explode all trees #24 MeSH descriptor: [Extramarital Relations] explode all trees #25 jealous*: ti #26 jealous*: ab #27 infidelity:ti #28 infidelity:ab #29 sexual affair:ti #30 sexual affair:ab #31 unfaithful*: ti #32 unfaithful*: ab #33 adultery:ti #34 adultery:ab #35 adulterer:ti #36 adulterer:ab #37 extramarital:ti #38 extramarital:ab #39 “controlling behavior”: ti #40 “controlling behavior”:ab #41 “controlling behaviour”:ti #42 “controlling behaviour”: ab #43 #23or#24or#25or#26or#27or#28or#29or#30or#31or#32or#33 or#34or#35or#36or#37or#38or#39or#40or#41or#42 #44 #22and#43
CINAHL (Cumulative Index of Nursing and Allied Health Literature)	S10 S1 OR S2 OR S3 OR S4 OR S5 OR S6 OR S7 OR S8 OR S9 S9 TI (domestic violence) or AB (domestic violence) S8 TI (partner* or spouse* or gender) N3 (violen*)) or AB (partner* or spouse* or gender) N3 (violen*)) S7 TI (batter* N3 (wom?n or wife or wives)) or AB(batter* N3 (wom?n or wife or wives)) S6 TI (abuse* N3 (wom?n or spouse* or partner* or wife or wives))or AB (abuse* N3 (wom?n or spouse* or partner* or wife or wives)) S5 (MH “Gender-Based Violence) S4 (MH “Dating Violence”) S3 (MH “Battered Women”) S2 (MH “Domestic Violence”) S1 (MH “Intimate Partner Violence”)	S23 S10 AND S22 S22 S11 OR S12 OR S13 OR S14 OR S15 OR S16 OR S17 OR S18 OR S19 OR S20 OR S21 S21 TI (extramarital) or AB (extramarital) S20 TI (“controlling behavior”) or AB (“controlling behavior”) S19 TI (“controlling behaviour”) or AB (“controlling behaviour”) S18 TI (adulterer) or AB (adulterer) S17 TI (adultery) or AB (adultery) S16 TI (unfaithful*) or AB (unfaithful*) S15 TI (“sexual affair”) or AB (“sexual affair”) S14 TI (infidelity) or AB (infidelity) S13 TI (jealous*) or AB (jealous*) S12 (MH “Jealousy”) S11 (MH “Extramarital Relations”)
Embase (Excerpta Medica Database)	Battered Woman/ OR domestic violence/ or intimate partner violence/ or family violence/ or battering/ OR (abuse adj3 (woman or women)) OR (abuse$ adj3 partner$) OR (abuse$ adj3 spouse$) OR ((wife or wives) adj3 batter$) OR ((wife or wives) adj3 abuse$) OR (violen$ adj3 partner$) OR (violen$ adj3 spous$) OR (violen$ adj3 (date or dating))	Jealousy/ or extramarital sexual intercourse/ or ((jealous*) or (infidelit*) or (“sexual affair*”) or (unfaithful*) or (adultery) or (adulterer) or (extramarital) or (“controlling behaviour*”) or (“controlling behavior*”))
Social Policy and Practice		
IBSS (International Bibliography of the Social Sciences)	(MAINSUBJECT.EXACT(“Domestic violence”) OR ((TI((“family violence”) or (“domestic violence”) or (“dat* violence”))) OR (AB((“family violence”) or (“domestic violence”) or (“dat* violence”))) OR ((TI((violen* near/3 (wom*n or partner* or spous* or wife or wives)))) OR (AB((violen* near/3 (wom*n or partner* or spous* or wife or wives)))) OR (AB((batter* near/3 (wom*n or partner* or spous* or wife or wives)))) OR (TI((batter* near/3 (wom*n or partner* or spous* or wife or wives)))) OR ((TI((abuse* near/3 (wom*n or partner* or spous* or wife or wives)))) OR (AB((abuse* near/3 (wom*n or partner* or spous* or wife or wives))))) OR (TI((abuse* near/3 (wom*n or partner* or spous* or wife or wives)))) OR (AB((abuse* near/3 (wom*n or partner* or spous* or wife or wives))))))	(MAINSUBJECT.EXACT.EXPLODE(“Adultery”) OR ((jealous*) or (infidelit*) or (“sexual affair*”) or (unfaithful*) or (adultery) or (adulterer) or (extramarital) or (“controlling behaviour*”) or (“controlling behavior*”))
Medline (Medical Literature Analysis and Retrieval System Online)	Battered Women/ OR Domestic Violence/ OR Spouse abuse/ OR intimate partner violence OR (abus$ adj3 partner$) OR (abus$ adj3 wom#n$) OR (abus$ adj3 spous$) OR ((wife or wives) adj3 batter$) OR ((wife or wives) adj3 abuse$) OR (violen$ adj3 partner$) OR (violen$ adj3 spous$) OR (violen$ adj3 (date or dating))	Jealousy/ or Extramarital relations/ or ((jealous*) or (infidelit*) or (“sexual affair*”) or (unfaithful*) or (adultery) or (adulterer) or (extramarital) or (“controlling behaviour*”) or (“controlling behavior”*))
PsycINFO (Psychological Information)	1 Battered Females/ 2 Domestic Violence/ 3 Intimate Partner Violence/ 4 (Partner Abuse.ab.) or (partner abuse.ti.) 5 (abuse$ adj3 wom#n).ab. or (abuse$ adj3 wom#n).ti. 6 (abuse$ adj3 spous$).ab. or (abuse$ adj3 spous$).ti. 7 (abuse$ adj3 partner$).ab. or (abuse$ adj3 partner$).ti. 8 (abuse$ adj3 (wife or wives)).ab. or (abuse$ adj3 (wife or wives)).ti. 9 (batter$ adj3 wom#n).ab. or (batter$ adj3 wom#n).ti. 10 (batter$ adj3 (wife or wives)).ab. or (batter$ adj3 (wife or wives)).ti. 11 (partner$ adj3 violen$).ab. or (partner$ adj3 violen$).ti. 12 (spous$ adj3 violen$).ab. or (spous$ adj3 violen$).ti. 13 (domestic violence).ab. or (domestic violence).ti. 14 (gender adj3 violen$).ab. or (gender adj3 violen$).ti. 15 or/1-14	16 jealousy/ 17 infidelity/ 18 extramarital intercourse/ 19 (unfaithfulness).ab. or (unfaithfulness).ti.20 (adultery).ab. or (adultery).ti. 21 (adulterer).ab or (adulterer).ti. 22 (sexual affair).ab. or (sexual affair).ti. 23 (controlling behaviour).ab. or (controlling behaviour).ti. 24 (controlling behavior).ab. or (controlling behavior).ti. 25 or/ 16-24 26 and/ 15, 24
Social Services Abstracts	MAINSUBJECT.EXACT.EXPLODE(“Spouse Abuse”) OR MAINSUBJECT.EXACT.EXPLODE(“Battered Women”) OR MAINSUBJECT.EXACT.EXPLODE(“Partner Abuse”) OR MAINSUBJECT.EXACT.EXPLODE(“Family Violence”) OR (SU.EXACT((“Family violence”)) OR SU.EXACT((“Partner Abuse”) OR (“Battered Women”)) OR (abuse NEAR/3 wom*n) OR (abuse NEAR/3 spouse*) OR (abuse NEAR/3 partner*) OR (wife NEAR/3 abuse*) OR (wives NEAR/3 abuse*) OR (wife NEAR/3 batter*) OR (wives NEAR/3 batter*) OR (women NEAR/3 batter*) OR (partner* NEAR/3 violen*) OR (spouse* NEAR/3 violen*) OR (gender NEAR/3 violen*) OR (“domestic violence”))	MAINSUBJECT.EXACT.EXPLODE(“Jealousy”) OR MAINSUBJECT.EXACT.EXPLODE(“Infidelity”) OR ((jealous*) or (infidelit*) or (“sexual affair*”) or (unfaithful*) or (adultery) or (adulterer) or (extramarital) or (“controlling behaviour*”) or (“controlling behavior*”))
Sociological Abstracts		
Web of Science	1 TI = ((family violence) or (domestic violence) or (dat* violence)) 2 AB = ((family violence) or (domestic violence) or (dat* violence)) 3 TI = ((violen* near/3 (wom*n or partner* or spous* or wife or wives))) 4 AB = ((violen* near/3 (wom*n or partner* or spous* or wife or wives))) 5 AB = ((batter* near/3 (wom*n or partner* or spous* or wife or wives))) 6 TI = ((batter* near/3 (wom*n or partner* or spous* or wife or wives))) 7 TI = ((abuse* near/3 (wom*n or partner* or spous* or wife or wives))) 8 AB = ((abuse* near/3 (wom*n or partner* or spous* or wife or wives))) 9 TI = ((abuse* near/3 (wom*n or partner* or spous* or wife or wives))) 10 AB = ((abuse* near/3 (wom*n or partner* or spous* or wife or wives))) 11 1 OR 2 OR 3 OR 4 OR 5 OR 6 OR 7 OR 8 OR 9 OR 10	12 TI = ((jealous*) or (infidelit*) or (“sexual affair*”) or (unfaithful*) or (adultery) or (adulterer) or (extramarital) or (“controlling behaviour*”) or (“controlling behavior*”)) 13 AB = ((jealous*) or (infidelit*) or (“sexual affair*”) or (unfaithful*) or (adultery) or (adulterer) or (extramarital) or (“controlling behaviour*”) or (“controlling behavior*”)) 14 12 OR 13

## Appendix B

Full quality assessment of included quantitative studies adapted from a validated tool [46] and the STROBE (Strengthening the Reporting of Observational Studies in Epidemiology) checklist [47].

**Table A2:** Quality assessment of included quantitative studies.

Author (Year)	External Validity						Internal Validity		Quality Assessment	
	Sample	Response Rate	Non-Response Bias	Missing Data	Study Subjects	Appropriateness	IPV Measure	Infidelity or RJ Measure	TOTAL (Out of 8)	Quality ^1^
Alan et al. (2016a)	0	1	0	0	1	0	0	0	2	★☆☆☆
Alan et al. (2016b)	0	0	0	0	1	0	0	0	1	★☆☆☆
Ansara and Hindin (2009)	0	1	1	1	1	1	1	1	7	★★★★
Chuemchit et al. (2018)	0	1	1	1	0	1	1	1	6	★★★☆
Conroy (2014)	1	1	1	0	1	1	0	0	5	★★★☆
Edelstein (2018)	1	1	1	0	0	1	1	0	5	★★★☆
Goetz and Shackelford (2009)	0	0	0	0	0	1	1	1	3	★★☆☆
Graham-Kevan and Archer (2011)	0	0	0	0	0	1	1	0	2	★☆☆☆
Guay et al. (2016)	0	0	0	0	1	1	1	0	3	★★☆☆
Kalokhe et al. (2018)	0	0	0	0	1	1	1	0	3	★★☆☆
Kerr and Capaldi (2011)	0	1	1	0	1	1	1	1	6	★★★☆
LaMotte et al. (2018)	0	1	1	0	1	0	1	1	5	★★★☆
Madsen et al. (2012)	0	0	0	0	1	1	1	1	4	★★☆☆
McKay et al. (2018)	0	1	0	0	1	1	1	0	4	★★☆☆
Messing et al. (2014)	0	1	0	0	1	1	1	0	4	★★☆☆
Paat et al. (2017)	1	0	0	1	1	1	1	0	5	★★★☆
Salwen and O’Leary (2013)	1	1	1	1	1	1	1	1	8	★★★★
Shrestha et al. (2016)	0	1	0	0	1	1	0	0	3	★★☆☆
Snead et al. (2019)	0	0	0	0	1	1	1	1	4	★★☆☆
Stieglitz et al. (2011)	0	0	0	1	1	1	0	0	3	★★☆☆
Stieglitz et al. (2012)	0	0	0	0	1	1	0	0	2	★☆☆☆
Toprak and Ersoy (2017)	1	0	0	0	1	1	1	0	4	★★☆☆
Townsend et al. (2011)	0	0	0	0	1	1	0	0	2	★☆☆☆
Ulibarri et al. (2010)	0	0	0	0	1	1	0	0	2	★☆☆☆
Wang et al. (2009)	1	1	0	0	1	1	0	0	4	★★☆☆

^1^ Studies were quality assessed on 8 core criteria that evaluated external and internal validity, scoring 0 or 1 for each, for a total possible score of 8. Studies scoring 7–8 points received ★★★★, studies scoring 5–6 points received ★★★☆, studies scoring 3–4 points received ★★☆☆ and studies scoring 2 points and lower received ★☆☆☆.

## Appendix C

**Table A3:** Measures of infidelity or romantic jealousy used in included quantitative studies.

Type of Measure	Measure Description	Author (Year)
Validated questionnaire or scale	Questionnaire developed from the WHO multi country study on women’s health and domestic violence. Controlling behaviours scale included: “got angry if female partner spoke with another man”, and “suspicious that female partner is unfaithful” [52].	Chuemchit et al. (2018)
	Authors developed the Perception of Aggression Scale: “What led you to…” Answer option includes: “because I was jealous” [57].	Guay et al. (2016)
	Conflict Tactics Scale - 2 Negotiation Subscale: “Extent of jealousy if spouse talks to men within family”, “Extent of jealousy if spouse talks to men outside family”. Also asked: “Engagement in sexual relations outside of spouse?” (“Yes” or “No”) [58].	Kalokhe et al. (2018)
	Modified Relationship Problem Scale [60].	LaMotte et al. (2018)
	Relationship Jealousy Scale (e.g., “How intense are your feelings of jealousy in your current relationship?”) 1-7 scale, “not at all” to “very” [61].	Madsen et al. (2012)
	Revised Conflict Tactic Scale (e.g., “How often does partner become jealous or possessive”, “you know you can count on your partner to remain faithful to you”). Likert scale [62].	McKay et al. (2018)
	Psychological Maltreatment of Women Scale. Jealousy measure is a 12 items scale derived from a factor analysis. Jealousy score was based on an average of the scores of the husband and wife [65].	Salwen and O’Leary (2013)
	Multidimensional Jealousy Scale. This includes 3 subscales for cognitive, emotional and behavioural Jealousy. Emotional jealousy assessed how “upset” partners would feel in response to various jealousy-evoking situations. Behavioural jealousy measured how often partners engaged in various protective behaviours (i.e., verbal attack of possible relationship competitors) and detective behaviours (i.e., going through their partner’s belongings) [67].	Snead et al. (2019)
Continuous or Likert scale questions developed by authors	Self-reported marital infidelity: “how many people they had sex with in the past 4 months/12 months” (including spouses). Perceived likelihood of partner having an affair: “My partner is probably having sex with someone else”. One to four scale, “strongly agree” to “strongly disagree” [53].	Conroy (2014)
	Partners past infidelities: “As far as you know, has your current partner had sexual intercourse with someone other than you since you have been involved in a relationship together?”, “As far as you know, has your current partner fallen in love with someone other than you since you have been involved in a relationship together?” Likelihood of committing an infidelity: “How likely do you think it is that your current partner will in the future have sexual intercourse with someone other than you, while in a relationship with you?” “How likely do you think it is that your current partner will in the future fall in love with someone other than you, while in a relationship with you?”. Ten point scale: definitely not/not at all likely–definitely yes/extremely likely. Male infidelity measurement not specified [55].	Goetz and Shackelford (2009)
	Frequency of disagreements stemming from partner’s jealousy, respondent’s commitment, partner’s commitment. Five point scale: “never argue about this” to “always argue about this” [56].	Graham-Kevan and Archer (2011)
	Number of physically abusive events/Frequency of disagreements stemming from partner’s jealousy, respondent’s commitment, partner’s commitment. Five point scale: “never argue about this” to “always argue about this” [68].	Stieglitz et al. (2011)
Multiple choice or binary [yes/no] questions	Controlling behaviour by the husband was assessed with two items: (a) “your husband or partner won’t let you wear certain things”, and (b) “your husband or partner tells you who you can spend time with” [51].	Ansara and Hindin (2009)
	Sexual jealousy: “Is your partner violently and constantly jealous of you? (For instance, does your partner say, ‘If I can’t have you, no one can?’)” [63].	Messing et al. (2014)
	“During the time you were together as a couple, do you think (the father of your child) ever cheated on you with another person after (child’s) birth” [64]?	Paat et al. (2017)
	Husband extramarital relationship (“Yes”, “No” or “Don’t Know”) [66].	Shrestha et al. (2016)
	“Do you think your partner has other sexual partners?” (“Yes” or “No”). “Think about the last 3 months, have you been in a sexual relationship with a woman whilst still having a sexual relationship with another?” (“Yes” or “No”) [71].	Townsend et al. (2011)
	“Since you have been together, has your spouse or steady partner had sex with another partner?” (“Yes” or “No”) [72].	Ulibarri et al. (2010),
	“How often do you feel jealous or quite insecure about your partner?” (“never/rarely” or “sometimes/often”). Same question asked to partner [73].	Wang et al. (2009)
Open-ended questions	“What are the reasons for domestic violence given by your husband” [49]?	Alan et al. (2016a)
	“What is the reason for violence in your opinion” [50]?	Alan et al. (2016b)
	“What are the worst arguments with your spouse in the past year, and throughout your marriage in other years” [69]?	Stieglitz et al. (2012)
Observational	Content analysis of court documents to determine motives of intimate partner homicide [54].	Edelstein (2018)
	Motives of femicides coded from legal files [70].	Toprak and Ersoy (2017)
Mix	Jealousy measure based on two self-report items on the couples interview, partner-reports on the couples interview and the partner issues checklist and one rating from the family and peer process code coder impressions [59].	Kerr and Capaldi (2011)

## Appendix D

Full quality assessment of included qualitative studies adapted from the CASP (Critical Appraisal Skills Programme) checklist [48].

**Table A4:** Quality assessment of included qualitative studies.

Author (Year)	Are the Results Valid?						What are the Results?			Will the Results Help Locally?	Quality Assessment	
	Was There a Clear Statement of the Aims of the Research?	Is a Qualitative Methodology Appropriate?	Was the Research Design Appropriate to Address the Aims of the Research?	Was the Recruitment Strategy Appropriate to the Aims of the Research?	Was the Data Collected in a Way That Addressed the Research Issue?	Has the Relationship Between Researcher and Participant been Adequately Considered?	Have Ethical Issues been Taken Into Consideration?	Was the Data Analysis Sufficiently Rigorous?	Is There a Clear Statement of Findings?	How Valuable is the Research?	Total (Out of 20)	Quality ^1^
Abreu et al. (2010)	2	2	1	2	2	1	2	1	1	2	16	★★★☆
Adinkrah (2014)	2	2	2	1	1	0	0	2	2	2	14	★★☆☆
Arpanantikul (2010)	2	2	2	2	2	1	2	2	2	2	19	★★★★
Bahadir-Yilmaz and Oz (2019)	2	1	2	1	1	0	1	1	2	0	11	★☆☆☆
Berg et al. (2010)	2	2	2	2	2	1	1	1	2	2	17	★★★☆
Boira et al. (2017)	2	1	1	1	1	0	0	2	2	1	11	★☆☆☆
Boyce et al. (2016)	2	2	2	2	2	0	1	2	2	2	17	★★★☆
Byun (2012)	2	1	2	1	1	0	1	1	1	1	11	★☆☆☆
Conroy et al. (2018)	2	2	2	2	2	0	0	1	2	2	15	★★★☆
Conroy et al. (2019)	2	2	1	2	2	1	2	1	2	2	17	★★★☆
Das et al. (2016)	2	2	2	1	2	0	2	0	1	1	13	★★☆☆
Fenton and Rathus (2009)	2	2	2	1	1	0	1	2	1	2	14	★★☆☆
Freysteinsdóttir (2017)	2	2	2	2	2	0	1	1	1	2	15	★★★☆
Gibbs et al. (2014)	2	2	1	1	2	0	2	2	1	2	15	★★★☆
Guruge et al. (2017)	2	2	1	1	2	0	2	2	1	2	15	★★★☆
Hatcher et al. (2016)	2	2	2	2	2	2	2	2	2	2	20	★★★★
Kyegombe et al. (2014)	2	2	2	2	2	1	2	2	2	2	19	★★★★
Nemeth et al. (2012)	2	2	2	2	1	0	2	2	2	2	17	★★★☆
Nhi et al. (2018)	2	2	2	2	2	0	2	2	2	2	18	★★★★
Nudelman et al. (2017)	2	2	2	2	2	0	2	2	2	1	17	★★★☆
Nur Hayati et al. (2013)	2	2	2	1	2	0	2	2	1	1	15	★★★☆
Orengo-Aguayo and Lawrence (2014)	2	2	2	2	2	0	2	2	2	2	18	★★★★
Paixão et al. (2014)	2	2	1	1	1	0	1	1	2	1	12	★★☆☆
Starmann et al. (2017)	2	2	2	2	1	0	1	1	2	2	15	★★★☆
Stith et al. (2011)	2	2	2	2	2	1	1	2	2	1	17	★★★☆
Varma et al. (2010)	2	2	2	2	2	0	2	1	2	1	16	★★★☆

**1** Studies were quality assessed on 10 core criteria that evaluated validity, ethics, data analysis, presentation of findings, and the value of the research. Studies were scored on a scale of 0–2, for a total possible score of 20. Studies scoring 18–20 points received ★★★★, studies scoring 15–17 points received ★★★☆, studies scoring 12–14 points received ★★☆☆ and studies scoring 11 points and lower received ★☆☆☆.
